# Tumor Necrosis Factor Receptor-Associated Factor 6 and Human Cancer: A Systematic Review of Mechanistic Insights, Functional Roles, and Therapeutic Potential

**DOI:** 10.7150/jca.90059

**Published:** 2024-01-01

**Authors:** Tingting Li, Zhe Lei, Lin Wei, Kai Yang, Jinhong Shen, Lin Hu

**Affiliations:** 1 State Key Laboratory of Radiation Medicine and Protection, School for Radiological and Interdisciplinary Sciences (RAD-X) and Collaborative Innovation Centre of Radiation Medicine of Jiangsu Higher Education Institutions, Soochow University, 199 Renai Road, Suzhou 215123, China.; 2 Institutes of Biology and Medical Sciences, Soochow University, Suzhou 215123, China.; 3 Department of Pathology, The First Affiliated Hospital of Soochow University, Suzhou 215006 Jiangsu, China.; 4 Shanghai Key Laboratory of Sleep Disordered Breathing, Department of Otolaryngology-Head and Neck Surgery, Otolaryngology Institute of Shanghai Jiaotong University, Shanghai Sixth People's Hospital Affiliated to Shanghai Jiaotong University School of Medicine, Shanghai 200233, China.

**Keywords:** TRAF6, Inflammatory, Tumor microenvironment, Tumor immunotherapy, Therapeutic target

## Abstract

Cancer imposes a substantial burden and its incidence is persistently increasing in recent years. Cancer treatment has been difficult due to its inherently complex nature. The tumor microenvironment (TME) includes a complex interplay of cellular and noncellular constituents surrounding neoplastic cells, intricately contributing to the tumor initiation and progression. This critical aspect of tumors involves a complex interplay among cancer, stromal, and inflammatory cells, forming an inflammatory TME that promotes tumorigenesis across all stages. Tumor necrosis factor receptor-associated factor 6 (TRAF6) is implicated in modulating various critical processes linked to tumor pathogenesis, including but not limited to the regulation of tumor cell proliferation, invasion, migration, and survival. Furthermore, TRAF6 prominently contributes to various immune and inflammatory pathways. The TRAF6-mediated activation of nuclear factor (NF)-κB in immune cells governs the production of proinflammatory cytokines. These cytokines sustain inflammation and stimulate tumor growth by activating NF-κB in tumor cells. In this review, we discuss various types of tumors, including gastrointestinal cancers, urogenital cancers, breast cancer, lung cancer, head and neck squamous cell carcinoma, uterine fibroids, and glioma. Employing a rigorous and systematic approach, we comprehensively evaluate the functional repertoire and potential roles of TRAF6 in various cancer types, thus highlighting TRAF6 as a compelling and emerging therapeutic target worthy of further investigation and development.

## Introduction

Cancer has emerged as a critical global health issue, positioned among the primary mortality drivers worldwide 1. The tumor microenvironment (TME) is a multifaceted and dynamic ecosystem, encompassing not only cancer cells but also various other cell populations 2. A complex interplay of biological pathways and mechanisms is crucial for both oncogenic transformation and maintenance of cancer phenotypes. Clinical and experimental evidence has revealed that inflammatory pathway activation can promote cancer development 3. Inflammatory pathways of two distinct types, intrinsic and extrinsic, are implicated in cancer progression. These pathways involve the initiation of critical transcription factors, including nuclear factor (NF)-κB, signal transducer and activator of transcription 3 (STAT3), and hypoxia-inducible factor (HIF-1α), within neoplastic cells 4. Importantly, tumor necrosis factor receptor-associated factor 6 (TRAF6), known for its involvement in NF-κB signaling, has been identified as a substantial contributor to neoplastic evolution, progression, and metastasis 5.

Within the TRAF protein family, TRAF6 distinguishes itself via a distinctive TRAF-C domain, allowing interaction with the cytoplasmic tail of receptors and other upstream entities. This unique structural feature confers specific physiological functionalities to TRAF6. Initially identified in 1986 for its specific role in regulating immunoglobulin κ light chain expression in B-cells, the transcription factor NF-κB was subsequently reported to participate in the signaling pathway activated by TRAF family proteins with E3 ubiquitin ligase activity. This finding revealed the downstream functional roles of these proteins within the receptor, pioneering a novel understanding of signal transduction mechanisms. In this context, the function of TRAF6 as an E3 ubiquitin ligase plays a key role in the assembly of K63-type polyubiquitin chains (K63-Ub chains). These K63-Ub chains serve as a scaffold for signal transduction complexes, activating downstream kinases 6, 7. During this catalytic process, TRAF6 attaches to ubiquitin via a thioester bond and transmits ubiquitin to relevant substrates, thus conveying signals within both immune and nonimmune cells. NF-κB activation is pivotal for various biological processes, including inflammation, innate and adaptive immunity, bone remodeling, appendage generation, and also for cancer initiation and progression. The activation of NF-κB occurs not only within malignant tumor cells but also within cellular components of the TME 8. These cells include, among others, M1/M2 macrophages, mast cells, dendritic cells, B cells, neutrophils, and T cells wherein the TRAF6-NF-κB pathway occupies a critical position. After NF-κB activation, cytokine production is initiated, further stimulating the activation of NF-κB in precancerous cells, resulting in the expression of genes associated with abnormal growth and culminating in the development of various cancers. Furthermore, NF-κB activation is intimately linked with bone metastasis because the receptor activator of nuclear factor κ B (RANK)-TRAF6-NF-κB pathway initiates osteoclastogenesis and the release of multiple growth factors from the bone microenvironment, promoting an environment conducive to cancer cell proliferation and colonization. Thus, TRAF6-induced NF-κB activation within the RANK and toll-like receptor (TLR) pathways is significantly implicated in the advancement and maintenance of cancer cells, particularly in cancers prone to bone metastasis, including breast 9, prostate 10, lung 11, and liver cancers 12.

At present, several studies are endeavoring to discover drugs that inhibit the activity of TRAF6-dependent enzymes in combination with radiation therapy or immunotherapy to control cancer development. Subsequent chapters will elucidate the involvement of TRAF6 in cancer, aiming to provide valuable perspectives on novel avenues for cancer therapeutic strategies.

## Overview of TRAF6 structure and its signal pathways

**Figure 1:**
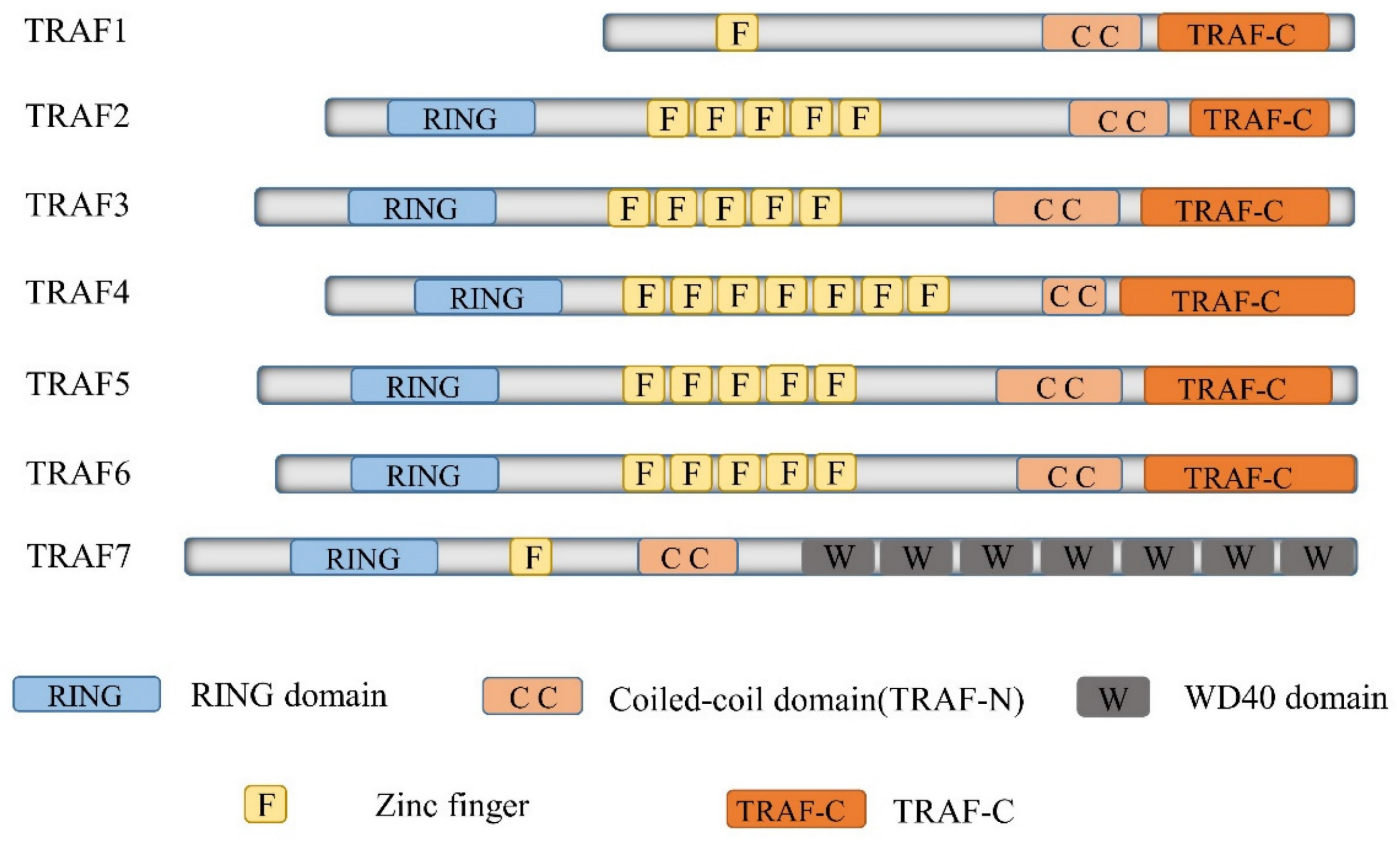
Delineates the structure of TRAF family members. The Tumor Necrosis Factor receptor-associated factor (TRAF) family represents a substantial class of intracellular adaptor proteins, encompassing seven members (TRAF1 through TRAF7). Excepting TRAF7, all TRAFs carry a remarkably conserved C-terminal motif, termed the TRAF domain. This motif, which includes approximately 200 amino acid residues, is bifurcated into TRAF-N and TRAF-C segments. Intriguingly, all TRAF proteins, with the exception of TRAF1, manifest an N-terminal RING-finger motif, succeeded by a sequence of five to seven zinc finger domains.

**Figure 2:**
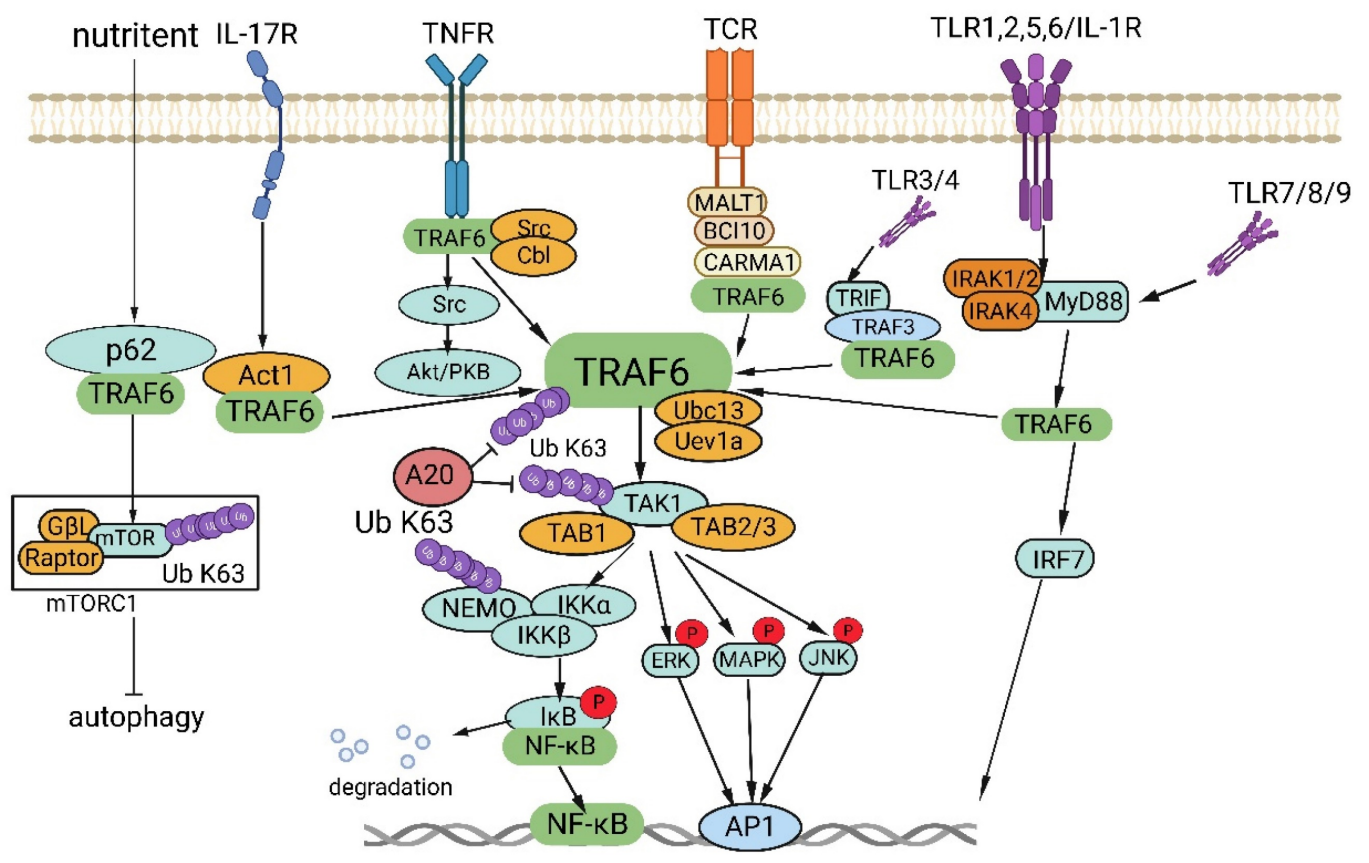
TRAF6 functions as a crucial adaptor protein in multifaceted signaling pathways. TRAF6 constitutes a pivotal component in various signaling pathways, including those involving IL-17R, the TNFR superfamily, TCR, and the TLR/IL-1 family. TRAF6 activation transpires via intermediary proteins such as Act1 in the IL-17R pathway and the MALT1-BCL10-CARMA1 complex in the TCR pathway. In certain scenarios, TLR family members, for example TLR3/4, employ TRIF to recruit TRAF3, thereby activating TRAF6. Upon its activation, TRAF6 associates with the ubiquitin-conjugating enzyme Ubc13 and its variant Uev1a, contributing to the formation of K63-linked polyubiquitin chains that subsequently signal and bind to lysine residues in target proteins, including inhibitory κB kinases (IKKγ, alternatively known as NEMO), TGF-β-activated kinase 1 (TAK1), IRAK1, and TRAF6 itself. TAK1, along with TAK1 binding proteins TAB1 and TAB2/3, form the TAB1-TAK1-TAB2/3 complex, which stimulates the IKKα/IKKβ/IKKγ complex, phosphorylates IκB, results in IκB degradation, and subsequently activates the transcription factor NF-κB. Furthermore, TRAF6 propels the activation of downstream interferon regulatory factor 7 (IRF7) through the TLR7/8/9-MYD88 pathway. Moreover, TAK1 instigates the activation of mitogen-activated protein kinase (MAPK) pathways, including the extracellular signal-regulated kinase (ERK) pathway, C-Jun N-terminal kinase (JNK) pathway, and P38 pathway. These MAPK pathways contribute to the activation of transcription factor AP-1 and are intricately involved in tumor initiation and progression. TRAF6 also operates through Akt and forms a complex with the Cbl family, contingent on Src kinase activity. In nutrient-stimulated cells, TRAF6 is recruited by P62 to activate mTORC1 through K63 ubiquitination. Lastly, TRAF6 modulates autophagy via its interaction with P62 and the activation of mTORC1.

TRAF6, a member of the TRAF protein family, is widely expressed across various species and has been detected in mammalian tissues. Presently, the TRAF family comprises seven known members, specifically TRAF1-7 (Figure 1). These TRAF proteins are characterized by distinct domains, including RING, zinc finger, coiled-coil, and TRAF-C. Except for TRAF1, each member of the TRAF family possesses a circular homolog domain at the N-terminal region, which serves as the central component for E3 ubiquitin ligase activity 13, 14. The ubiquitin-proteasome system (UPS) comprises three primary enzymes: ubiquitin activase (E1), ubiquitin-binding enzyme (E2), and ubiquitin-protein ligase (E3) 15. TRAF6 binds to a specific substrate and collaborates with E1, E2, and ubiquitin proteins to bind to the substrate. Ubiquitination regulates intracellular signal transduction through two primary types of ubiquitination: Lys48 (K48)-linked polyubiquitin for proteasome-targeted protein degradation and K63-Ub chains for posttranslational modifications without inducing proteasome degradation 16, 17. TRAF6, along with the ubc13-uevla E2 complexes, generates K63-Ub chains that selectively bind to the ubiquitin-binding domains and recruit proteins 18. The TRAF-C domain of TRAF6 exhibits distinct binding preferences compared to other TRAF proteins. It recognizes specific amino acid sequences, including X-X-P-X-E-X-X acidic or aromatic motifs 19, which differ from the P-X-Q-X-T motifs recognized by TRAF2, TRAF3, and TRAF5 20, 21. Studies on TRAF6 structural features have revealed valuable insights regarding its effects on cellular signal transduction (Figure 2). TRAF6 is involved in several signaling pathways, including the TLR/interleukin (IL)-1 family, tumor necrosis factor receptor superfamily, IL-17R, and T cell receptor (TCR) pathways. The activation process involves intermediary proteins such as Act1 for IL-17R and the MALT1-BCL10-CARMA1 complex relevant to TCR signaling. Under particular conditions, TRAF6 stimulation occurs via associations with TLR family constituents, such as TLR3/4, which engage TRIF, recruit TRAF3, and subsequently activate TRAF6 22-24. On activation, TRAF6 forms assemblies with Ubc13 and its variant Uev1a. It then facilitates signal transduction by attaching K63-Ub chains to lysine residues on various target proteins, including inhibitor of κB kinase (IKK)γ (also known as NEMO), transforming growth factor-β-activated kinase 1 (TAK1), interleukin-1 receptor-associated kinase 1 (IRAK1), and TRAF6 itself 25, 26. When TAK1 interacts with TAB1 and TAB2/3 proteins, the TAB1-TAK1-TAB2/3 complex is formed, resulting in the activation of the IKKα/IKKβ/IKKγ complex and subsequent phosphorylation of inhibitor of κB (IκB). This phosphorylation event induces the IκB degradation and the subsequent activation of the transcription factor NF-κB 27, 28.

Disrupted regulation of the NF-κB pathway frequently manifests in cancer, and genetic alterations affecting various regulatory components of NF-κB have been detected in specific lymphatic malignancies 29. Activation of NF-κB is also commonly observed in solid tumors and is deemed pivotal for tumor advancement. Significantly, activation of NF-κB has been implicated in conferring resilience against chemotherapy and radiation therapy in oncological treatment 30. In addition, TRAF6 is involved in the activation of interferon regulator 7 (IRF7) through the TLR7/8/9-MyD88 pathway 31. Further, TAK1 instigates the activation of MAPK pathways, encompassing the ERK, JNK, and P38 pathways 32. The activation of these MAPK pathways culminates in the activation of the transcription factor AP-1, which is intimately connected with tumor genesis and manifestation.

TRAF6 also functions in stimulating protein kinase B (Akt) via RANK and CD40 in various cell types. The engagement between RANK or CD40 and the Cbl family of scaffold proteins, along with TRAF6, is orchestrated by Src kinase activity. Cbl facilitates the recruitment of phosphoinositide 3-kinase (PI3K) to the receptor complex, resulting in Akt activation, which subsequently influences cellular processes, including glucose metabolism, cell proliferation, apoptosis, transcription, and cell migration 33, 34. Furthermore, recent research has revealed context-specific mechanisms of TRAF6 involving interactions with regulatory proteins and the microRNA (miRNA) regulation at the mRNA level. In amino acid-stimulated cells, P62 recruits TRAF6, triggering mTORC1 activation via K63 ubiquitination 35. Moreover, TRAF6 regulates autophagy by interacting with P62 and activating mTORC1, thereby significantly contributing to cancer cell proliferation.

## TRAF6 in cancers

Maintaining homeostasis requires a delicate balance between cell apoptosis and proliferation. The genesis and progression of neoplasms are not solely governed by the proliferation and differentiation of neoplastic cells but also significantly by their apoptosis. An increase in TRAF6 expression has been reported in the majority of human malignant neoplasms 36-39. As an E3 ubiquitin ligase, TRAF6 contributes to protein degradation and acts as a bridging factor in signaling pathways involving both K48 and K63 ubiquitin chains. It modulates cell proliferation, differentiation, apoptosis, and negatively regulates autophagy via various signaling pathways, primarily the NF-κB signaling pathway. This association can be attributed to the widespread overexpression of TRAF6 across various types of human cancer, arising from the abnormal proliferation, differentiation, and apoptosis of neoplastic cells. Simultaneously, TRAF6 overexpression shows a strong correlation with tumor stage and can serve as a prognostic marker for overall survival 40. Regulated by miRNAs, TRAF6 is involved in cellular invasion and migration, promoting oncogenesis. In the following sections, we provide a comprehensive overview of the current understanding regarding the role of TRAF6 in gastrointestinal neoplasms, urogenital malignancies, myoid cancers, breast cancer, and other cancer categories.

## TRAF6 in gastrointestinal cancers

### Colorectal cancer

Chronic inflammation plays a pivotal role in the development of colitis-associated cancer. This stems from the induction of oxidative stress, which triggers DNA damage and subsequently activates genes linked to carcinogenesis. Furthermore, it hampers the functioning of tumor-suppressor pathways. Colorectal cancer (CRC), one of the most prevalent malignancies, has become a focal point in scientific research. The primary treatment options currently encompass surgical interventions and chemotherapy. Nevertheless, patients diagnosed with advanced-stage CRC face a significant risk of recurrence after surgery, and the effectiveness of chemotherapy is frequently compromised due to drug resistance. Additionally, there is evidence suggesting that TRAF6 enhances the resistance of colon cancer cells to chemotherapeutic agents such as fluorouracil and etoposide 41.

The expression of TRAF6 has been observed to correlate with a range of clinicopathological characteristics. A significant association has been observed between TRAF6 expression and Duke's stage, differentiation capacity, and lymph node metastasis in colorectal cancer patients (*P*<0.05). Yet, no significant correlation was found concerning gender and age (*P*>0.05). Moreover, elevated TRAF6 expression levels were inversely related to the 5-year survival rate. Patients exhibiting heightened TRAF6 expression manifested significantly lower survival rates compared to those with attenuated TRAF6 expression (*P*<0.05). Therefore, TRAF6 has been proposed as a prognostic indicator for colorectal cancer 42. TRAF6 exerts a significant role in the pathogenesis of colon cancer. KDM4B, an enzyme involved in AKT and NF-κB activation, has been observed to modulate GLUT1 expression via the AKT signaling pathway, thereby promoting glucose uptake and ATP production 43.

TRAF6 acts as a mediator between hypoxia signaling and the inflammatory cascade by regulating the NF-κB pathway 44, 45. It interacts with HIF-1α, facilitating its K63-linked polyubiquitination and promoting tumor angiogenesis 46. In MC-38 tumor cells, TRAF6 influences colon cancer development under hypoxic conditions 47. The hypoxia-responsive TRAF6/ATM signaling axis enhances HIF-1α activation, promoting tumorigenesis, and metastasis 48. ARC modulates intestinal homeostasis and forestalls Inflammatory Bowel Disease (IBD) through TRAF6 ubiquitination and NF-κB activation in T cells 49. The transgenic expression of tRXRα in murine models expedites the progression of Colitis-Associated Cancer (CAC) through the induction of IL-6 production and activation of STAT3 50. TRAF6 engages in the LPS-NF-κB-VEGF-C signaling pathway, influencing lymphangiogenesis via ubiquitination. Significantly, TRAF6 124mut curtails tumor growth both *in vitro* and *in vivo* 51.

TRAF6 instigates the activation of the TRAF6-NF-κB/AP-1 signaling pathway following nuclear translocation. This triggers behavioral changes in CRC cells 52. The interaction between STX2 and TRAF6 amplifies the activity of the NF-κB pathway, leading to elevated STX2 expression and creating a positive feedback loop that fosters CRC metastasis 53. The formation of the TAK1-TAB3-TRAF6 complex involving TAB3 facilitates CRC metastasis by activating the NF-κB pathway 54. TRIM25 impedes EZH2 ubiquitination via TRAF6, maintaining stem cell properties and promoting resistance to oxaliplatin in CRC cells 55. A nonsynonymous variant of IRAK2, rs35060588 (encoding R214G), has been identified as a participant in NF-κB signaling and TLR-mediated cytokine induction. This variant leads to reduced TRAF6 ubiquitination, a crucial element in signal transduction 56.

Beyond these functions, TRAF6 has been observed to stimulate autophagy in CRC. Meanwhile, TRAF6 regulates the abundance of RIPK1, inhibits RIPK1/RIPK3/MLKL necrosis signaling pathways and affects the progression of colorectal cancer 57. At the mechanistic level, TRAF6 interacts with MAP1LC3B/LC3B through its LC3 interaction region “YxxL” and catalyzes K63-linked polyubiquitination of LC3B. This ubiquitination facilitates the formation of the LC3B-ATG7 complex, a pivotal component in the selective autophagy degradation of CTNNB1 58. In colon cancer, NLR family CARD domain containing 3 (NLRC3) interacts with PI3K, restraining the activation of the downstream molecule AKT. This influence extends to the phosphorylation of mTOR, FoxO3a/O1, and the expression of cMyc, thereby impacting tumorigenesis 59. To summarize, these findings underscore the multifaceted roles of TRAF6 within the context of CRC.

A mounting body of evidence emphasizes the diverse engagement of microRNAs (miRNAs) in the pathogenesis of CRC. Importantly, an overexpression of miR-146b-5p in HT29 and SW620 cells was found to diminish TRAF6 expression. By interacting with TRAF6, miR-146b-5p targets and inhibits TRAF6 expression, thereby fostering the initiation and progression of CRC 60. Furthermore, specific ablation of miR-146a in bone marrow cells facilitated CRC development. This miRNA constrained prostaglandin E2 (PGE2) production in the tumor microenvironment, leading to the mitigation of intestinal inflammation and CRC progression by restricting myeloid cell-mediated IL-17 production or by directly targeting IL-17R-induced COX-2 and inhibiting the enzyme PTGES2 responsible for PGE2 synthesis 61. However, it is worth noting that the signaling landscape may exhibit substantial variations in different CRC cells, resulting in diverse regulatory outcomes and functional disparities within the same tissue. For instance, while miR-146a inhibited the migratory capacity of SW480 cells, it amplified the migratory capacity of Caco-2 cells 62. Additionally, miR-140 modulated the secretion of inflammatory cytokines, including IL-6, COX-2, TNF-α, VEGF-C, and MMP-7, in CRC cells in response to LPS stimulation by targeting TRAF6. Binding of miR-140 to the 3ʹ untranslated region (UTR) of TRAF6 mRNA resulted in TRAF6 mRNA degradation and reduced TRAF6 protein expression, ultimately attenuating the NF-κB/C-Jun signaling pathway activity and influencing the expression of associated genes 63. Taken together, these contrasting discoveries highlight the intricacies embedded in cancer management and underscore the imperative to elucidate the specific functional roles of disparate molecular mechanisms related to TRAF6 in the context of CRC.

### Gastric cancer

Gastrointestinal cancer (GC), a prevalent and lethal neoplasm affecting the digestive system, ranks third globally regarding both incidence and mortality across all cancer types. Abnormal TRAF6 expression has been detected in GC tissues, promoting the proliferation and migration of GC cells. Conversely, silencing TRAF6 has demonstrated the ability to attenuate these effects. Interestingly, TRAF6 appears to hinder differentiation while promoting stemness and the EMT process. Through the analysis of 701 differentially expressed genes between wild-type and TRAF6 knockout groups, potential molecular players linked to cell proliferation and migration, including mitogen-activated protein kinase (MAPK), forkhead box O, and IL-17, have been identified 64. Han et al. conducted an investigation that revealed high expression of TRAF6 in 58.9% (53 of 90) of GC cases. They observed that higher expression of TRAF6 is significantly correlated with advanced N stage, pathological stage, and poor prognosis of GC. However, TRAF6 expression did not emerge as an independent predictor of poor prognosis in GC 65. *Helicobacter pylori* infection is widely known as the primary cause of GC. Mechanistically, *H. pylori* triggers the transient activation of IKK complexes via a type IV secretory system-dependent and CagA-independent pathway. This IKK complex is stabilized and tightly regulated by IKKα, IKKβ, MEKK3, and TAK1 66. CagA, a crucial virulence factor in *H. pylori*, functions as a negative regulator of the MAPK and NF-κB signaling pathways. CagA interacts with host cell tyrosine phosphatase SHP1, initiating the recruitment of SHP-1 to TRAF6 and inhibiting K63-linked ubiquitination of TRAF6. This interference disrupts downstream signal transduction, providing a potential molecular basis for therapies targeting microbial infections 67. Both NF-κB and β-catenin signaling pathways play key roles in GC development by inducing double-strand breaks, defects in the mitotic checkpoint, deregulation of homologous recombination-mediated DNA double-strand break repair, and alterations in DNA repair enzymes. These events result in random gene modifications, activating oncogenes and inactivating tumor suppressor genes, ultimately leading to GC progression 68. Gunter Maubach et al. demonstrated that TRAF6 and TRAF2 serve as binding partners of TIFA, thereby activating both classical and alternative NF-κB signaling in H. pylori-infected gastric epithelial cells 69. Autophagy regulation and its impact on GC tumorigenicity have been linked to TRAF6. MiRNAs and their role in posttranscriptional gene regulation in the context of H. pylori infection are subjects of ongoing research. MiR-146a, for instance, appears to mitigate inflammation by targeting IRAK1 and TRAF6, modulating NF-κB activity 70. Furthermore, the positive correlation between the expression of miR-146a and IL-17a in H. pylori-infected human gastric mucosa suggests the regulatory role of miR-146a on IL-17A-induced proinflammatory cytokines 71. Further investigations have demonstrated that decreased miR-146a-5p expression is associated with invasion, metastasis, venous vascular invasion, size, and differentiation of GC. In contrast, the expression of long noncoding RNA HCG18 is increased in GC. HCG18 overexpression inhibits miR-146a-5p and upregulates the expression of TRAF6 and P65. This dysregulation of the miR-146a/TRAF6 axis by HCG18 promotes the progression of GC, indicating that HCG18 is a potential therapeutic target for GC 72. In summary, TRAF6 emerges as a critical factor in promoting the development of GC. Targeting TRAF6 holds promise for precision treatment strategies in GC management.

### Pancreatic cancer

Pancreatic cancer (PC) is an exceptionally heterogeneous disease 73, presenting formidable treatment challenges due to its chemoresistance, high aggressiveness, metastatic propensity, and dismal prognosis 74. The involvement of TRAF6, a pivotal regulator of NF-κB signaling, in the initiation and progression of PC has garnered substantial attention. NF-κB activation in PC plays a pivotal role in angiogenesis, metastasis, anti-apoptosis, and resistance to gemcitabine chemotherapy. Conversely, its inhibition can effectively reverse these deleterious effects 75. Clinical specimens from patients with PC frequently exhibit elevated TRAF6 levels. *In vitro* experiments have elucidated that heightened TRAF6 levels within PC cells augment cellular proliferation and migration, whereas reducing TRAF6 compromises tumorigenic potential in *in vivo* models. Mechanistically, TRAF6 activation triggers anti-apoptotic pathways and elevates the expression of an array of elements engaged in orchestrating cell cycle progression and cellular migration 76. Additionally, ubiquitin-specific protease 4 has emerged as a stabilizer of TRAF6 at the protein level, facilitating the initiation of NF-κB signaling and promoting proliferation, migration, and invasion of PC cells 77. Furthermore, TAK1 protein levels can be differentially regulated through ubiquitination of Yes-associated protein (YAP) and transcription coactivator (TAZ) via K63 and K48 ubiquitin linkages facilitated by PDZ binding domains and ubiquitous TRAF6 and ITCH. This regulation prevents proteasomal degradation and inhibits the carcinogenic effects of YAP/TAZ in PC 78. Research substantiates the regulatory role of TRAF6 in the oncogenic Hippo-YAP pathway, highlighting its facilitative role in the ubiquitination and subsequent degradation of MST1 within the context of PC. This establishes a crucial link between inflammatory processes and tumorigenesis 79. Inflammatory conditions, such as pancreatitis, are well-established risk factors for PC development. The overexpression of TLR4, NOD1, and TRAF6 genes, combined with the decreased expression of MyD88-encoding genes in peripheral leukocytes of patients with PC, may be implicated in chronic inflammation and tumor progression via the upregulation of inherent antibacterial responses, including those triggered by lipopolysaccharide (LPS) 80. In cases of acute pancreatitis, TRAF6 serves a protective function by inhibiting apoptosis of acinar cells. However, LPS-induced upregulation of Socs1 and Socs3 promotes TRAF6 degradation via hyperubiquitination, subsequently exacerbating pancreatic inflammation, manifesting in mild-to-severe intensity 81. Pancreatic ductal adenocarcinoma (PDAC), the most prevalent pancreatic malignant tumor, originates from the pancreatic ductal epithelium. CYB5A has been reported to induce autophagy, and network analysis of the autophagy pathway indicates an interaction between CYB5A and TRAF6, suggesting the involvement of TRAF6 in the CYB5A-mediated autophagy pathway 82. Genetic abnormality complexities and subsequent activation of signaling pathways contribute to high inter-tumor and intra-tumor heterogeneity, which are key characteristics of PDAC, leading to various autophagy-related survival or death events 73. Chemoresistance significantly affects the prognosis of patients with PDAC. The regulatory axis of miR-146a-5p and TRAF6 has been discerned as a key influencer of chemoresistance in pancreatic malignancies, primarily via the modulation of p-glycoprotein 83. Furthermore, long noncoding RNA-PLACT1 has been reported to contribute toward increasing pancreatic cancer progression by activating the NF-κB signaling pathway by reducing levels of the NF-κB inhibitor IκBα 84. Remarkably, it has been documented that the concurrent application of proteasome inhibitors and ionizing radiation (IR) induces autophagic cell death via TRAF6 downregulation, suggesting a potential novel approach to enhance the radiosensitivity of PC 85. In conclusion, investigations into the mechanisms involving TRAF6 in PC provide valuable insights for the treatment of PC.

### Esophageal carcinoma

The incidence of esophageal carcinoma (EC) exhibits an upward trajectory, and similar to many cancers, it progresses through distinct stages. Prolonged activation of specific signal transduction pathways stands as a pivotal factor in the initiation and progression of EC 86. Investigations have revealed a noteworthy amplification of TRAF6 expression in EC tissues. Patients displaying elevated TRAF6 expression often exhibit significantly shorter survival durations than those exhibiting reduced expression. The oncogenic impact of TRAF6 in EC is mediated via the upregulation of asparaginyl endopeptidase and matrix metalloproteinase 2. Furthermore, a pronounced correlation exists between high TRAF6 expression and short-term recurrence in patients with EC, suggesting the potential of TRAF6 as a biomarker or therapeutic target for EC management 87. A study by Ma and colleagues showed that TRAF6 leads to excessive activation of NF-κB in EC109 cells, contributing to the inhibition of apoptosis and promotion of cell proliferation 88. It is worth noting that EC comprises two primary histological subtypes: esophageal adenocarcinoma (EAC) and esophageal squamous cell carcinoma (ESCC), with the latter being predominating globally 89. Research by Han et al. has demonstrated that TRAF6 enhances the migration of ESCC cells and immortalized esophageal epithelial cells through the modulation of Ras signaling 90. Mechanistically, TRAF6 has been reported to modulate the expression of critical genes encoding proteins such as c-Jun, c-Myc, and Bcl-2, along with the caspase activation. Considering c-Jun, c-Myc, and Bcl-2 are established targets of the NF-κB signaling pathway, these findings suggest that the biological role of TRAF6 in ESCC likely involves the regulation of this pathway 91. In the context of EAC, TLR4 stimulation directly affects the growth and proliferation of both benign and malignant esophageal cells via the MyD88-TRAF6-NF-κB pathway 92. Research indicates that simvastatin and atorvastatin may prevent EAC tumor growth by inhibiting the TLR4 pathway 93. Notably, within the context of ESCC, it has been observed that cancer cells induce elevated levels of miR-146a in peripheral blood mononuclear cells (PBMCs), resulting in the downregulation of IRAK1 and TRAF6. This, in turn, attenuates the synthesis of components related to NF-κB signaling. Such a mechanism may diminish the ability of ESCC-PBMCs to generate and secrete proinflammatory mediators, aiding the tumor in devising immune evasion strategies, ultimately fostering tumor growth and progression 94. In summary, TRAF6 plays a significant role in the pathogenesis of EC.

### Hepatocellular carcinoma

Hepatocellular carcinoma (HCC), the predominant form of primary liver cancers, has exhibited a concerning global increase in prevalence. Prominent risk factors for liver cancer include chronic infections with hepatitis B or hepatitis C virus, alcoholic liver disease, and nonalcoholic fatty liver disease 95. Research conducted by Li and colleagues has underscored the significant role of TRAF6 in facilitating HCC progression. Diminished TRAF6 expression has been directly correlated with reduced patient survival rates 96. TRAF6 appears to promote metastasis and HCC progression by influencing cell growth and apoptosis, suggesting its potential as both a predictive and therapeutic biomarker for HCC 97. AKR1C3, an enzyme from the aldoketo reductase family, modulates NF-κB activity in HCC cells by regulating TRAF6 and inducing self-ubiquitination. This activation of NF-κB results in the release of proinflammatory factors (IL1β, IL6, and TNFα), enhances STAT3 phosphorylation, and ultimately increases tumor cell proliferation and invasion 98. The long noncoding RNA, SNHG16, is upregulated in HCC and has been found to bind and inhibit miR-605-3p, thereby augmenting TRAF6 expression. This sustained TRAF6 activity maintains an active NF-κB pathway, expediting EMT and metastasis in HCC 99. Research suggests that growth arrest and GADD34 mitigate TRAIL-induced apoptosis in HCC cells through TRAF6 and ERK-mediated stabilization of the Bcl-2 family member MCL-1 100. Recent findings have identified enhanced TRAF6 expression as a promoting factor of HCC by increasing the expression and stability of c-Myc 101. Inhibitors of TRAF6 have shown promise in inhibiting HCC growth, and combining a TRAF6 inhibitor with a PD-1 blocker has yielded enhanced therapeutic outcomes 102. TMBPS, a novel inhibitor, inhibits the proliferation of hepatocellular carcinoma HepG2 cells in a time- and dose-dependent manner. TMBPS directly binds to and reduces the levels of TRAF6, resulting in cell cycle arrest in the G2/M phase by deactivating the protein kinase B (Akt) and ERK1/2 signaling pathways. Moreover, TMBPS induces cell apoptosis by activating the p38/MAPK signaling pathway 103. A comprehensive understanding of the role of TRAF6 in HCC pathogenesis and tumor progression is crucial for the development of effective treatment modalities for HCC.

## TRAF6 in lung cancer

Extensive research has provided evidence of the potential oncogenic role of TRAF6 in lung cancer 104. This is substantiated by increased levels of TRAF6 observed in several lung cancer cell lines, including A549, HCC827, NCI-H292, and 95-D, as well as human bronchial epithelial cells. Conversely, the reduction of TRAF6 has been associated with decreased cell survival rates, suppressed cell proliferation and invasion, and enhanced cell apoptosis 105. Genetic abnormalities, such as somatic mutations and copy number alterations resulting from chromosome deletions or amplifications, are pivotal in the pathogenesis of lung cancer. A comprehensive analysis of chromosome amplification in lung cancer has identified TRAF6 as a critical oncogene in RAS-mediated tumorigenesis. Overexpression of TRAF6 in this context initiates NF-κB activation, anchorage-independent growth, and tumorigenesis 106. TRAF6 also plays a vital role in tumor glycolysis by mediating Akt ubiquitination, leading to enhanced Akt activation and increased HIF-1α-mediated transcription of hexokinase-2, thereby increasing tumor glycolysis in primary non-small cell lung cancer (NSCLC) 107. Furthermore, extensive research has focused on the involvement of TRAF6 in autophagy induction during lung cancer development. Autophagy, a process involving various proteins, prominently features BECN1, a central component of the BECN1-PIK3C3-PIK3R4 complex crucial for autophagy initiation 108. The molecular regulation of BECN1 phosphorylation by AMP-activated protein kinase (AMPK) and ubiquitination by TRAF6 may crucially contribute to autophagy induction, especially when stimulated by TLR4. This facilitates lung cancer cell migration and invasion 109. Studies on USP15 have indicated that autophagy-mediated TRAF6-BECN1 signaling negatively affects lung cancer progression 110. Additionally, TRIM59 has been implicated in autophagy regulation in NSCLC, affecting BECN1 transcription and ubiquitination 111. Stratifin promotes the assembly of the TRAF6-BECN1-VPS34 complex and enhances BECN1 ubiquitination, thereby facilitating tumor progression through autophagy induction 112. Furthermore, autophagy, stimulated by either TLR4 or TLR3 activation, promotes the generation of various cytokines by elevating TRAF6 ubiquitination, which consequently fosters lung cancer cell invasion and migration 113. Transforming growth factor-β1 has been identified as a modulator of autophagy through Smad4 and the TAK1-TRAF6-P38 MAPK pathways, influencing AMPK-dependent phosphorylation of ULK1 at S555 114. Altogether, these empirical findings suggest that manipulating autophagy could potentially serve as a viable therapeutic approach in the management of lung cancer.

## TRAF6 in urogenital cancers

Renal cell carcinoma (RCC), a common malignancy within the urological spectrum, is largely dominated by the clear cell renal cell carcinoma (ccRCC) subtype, which constitutes the majority of diagnosed instances 115. It has been established that TP53INP2 incites apoptosis in ccRCC via the Caspase-8/TRAF6 pathway, operating independently from the autophagy-mediated pathway 116. Prostate cancer (PCa) exhibits a high propensity for bone metastasis, wherein RANKL interacts with its receptor RANK via TRAF6, IKK, and p65-mediated cascades, resulting in NF-κB activation and subsequent osteoclast development. Inhibition of NF-κB can impede the growth of metastatic prostate tumors by targeting the essential osteolytic component 10. TRAF6 plays a critical role in regulating Wnt3a-induced β-catenin activity and the activation of Wnt3a target genes associated with PCa progression 117. Previous studies have demonstrated that TRAF6 activates the AKT pathway, promoting PCa advancement and invasion both *in vitro* and *in vivo*, highlighting its significant contribution to cancer progression 118-120. TGF-β exploits TRAF6, an E3 ligase, to activate the kinase TAK1, leading to PCa cell death 121. The ubiquitination of EZH2 in human PCa is mediated by TRAF6 under the auspices of SKP2, and the suppression of tumorigenesis is achieved via the decrease in EZH2 levels facilitated by the deletion of SKP2 122. TRAF6 imparts polyubiquitination to the PI3K regulatory subunit p85α, which fosters the assembly of a complex involving the TGF-β type I receptor (TβRI) and p85α, subsequently triggering the activation of PI3K and AKT 123. Intriguingly, the activation of Pak1 triggered by TGFβ1 is not reliant on the canonical Smad2-mediated pathway, but instead, it hinges on a non-canonical pathway steered by TRAF6. The inhibition of TGFβ1-induced EMT and invasion in PCa can be achieved by directing IPA 3 towards Pak1 124. In conclusion, TRAF6 represents a crucial target for inhibiting PCa progression and improving the prognosis of PCa patients. In other urogenital cancers, such as advanced cervical cancer, DRAK1 suppresses pro-inflammatory signaling by promoting TRAF6 degradation, thereby inhibiting inflammatory signaling-mediated tumor growth and metastasis 125. MiR-146a regulates Th17 cell differentiation through NF-κB signaling by targeting TRAF6, impacting cervical cancer cell growth and apoptosis 126. However, he elucidation of TRAF6's involvement in ovarian cancer continues to be comparatively underexplored.

## TRAF6 in breast cancer

While the role of TRAF6 in human breast cancer is not yet fully understood, some studies suggest its potential oncogenic activity. As an E3 ubiquitin ligase, the mediation of AKT ubiquitination and subsequent phosphorylation by TRAF6 has emerged as a critical determinant in cancer progression 127. TRAF6 is known to undergo self-ubiquitination and activation in response to hypoxia, leading to the formation of MUb-H2AX at K119/120. This process governs γH2AX formation and the induction of HIF1α target genes, which can influence cancer development 128. Significantly, TRAF6 is suspected to interact with the accessory molecule TAK1 through the AKT/GSK3β signaling pathway, potentially promoting cell proliferation 129. Remarkably, in the context of breast cancer and osteoclast-mediated bone degeneration, a small molecular inhibitor of TRAF6, 6877002, has shown efficacy in reducing metastasis, osteolysis, and osteoclast formation in osteotropic breast cancer models of both human and murine origin 130. Furthermore, in triple-negative breast cancer, the downregulation of TLR5 has been observed to enhance tumor aggressiveness and promote EMT via the TRAF6 and SOX2 pathways 131. In summary, TRAF6 appears to play a pivotal role as a molecular intermediary in breast cancer. However, further in-depth research is essential to fully elucidate the intricacies of its underlying mechanisms in breast cancer progression and development.

## TRAF6 in Myeloid neoplasms

NF-κB activation has been associated with bone metastasis, particularly through the RANK-TRAF6-NF-κB signaling pathway. This pathway plays a crucial role in orchestrating a microenvironment favorable for cancer cell proliferation and colonization within the bone structure. Therefore, TRAF6 is assumed to play a pivotal role in myeloid malignancies, including myeloproliferative neoplasms, acute myeloid leukemia, myelodysplastic syndromes, and related precursor cell neoplasms. In particular, multiple myeloma, a malignant neoplasm originating from plasma B cells in the bone marrow, has been a subject of study. Liu et al. revealed that blocking TRAF6 activity is a potential treatment for MM and associated bone diseases 132. *In vitro* studies have shown that increased TRAF6 expression is positively associated with enhanced myeloma cell proliferation. Inhibiting TRAF6 using small interfering RNA effectively counters this proliferative effect. The TRAF6-NF-κB signaling pathway in myeloma cells and bone marrow stromal cells presents a promising target for prognostic assessment and therapeutic intervention in MM 133. In patients with chronic myeloid leukemia (CML), research has revealed grancalcin as a catalyst for K63-linked ULK1 ubiquitination via TRAF6 activation. This process induces autophagy, contributing to the development of resistance to imatinib treatment in patients with CML. Moreover, a study by Fang et al. revealed the involvement of TRAF6 in mediating autophagy in myeloid leukemia cells after proteasome inhibition by bortezomib 134. Interestingly, the absence of TRAF6 in preleukemic cells has been associated with dominant myeloid leukemia and MYC-dependent stem cell characteristics. In patients with myeloid malignancies, TRAF6 inhibition has been observed, suggesting its potential contribution to the onset of acute leukemia. Mechanistically, TRAF6 ubiquitinates MYC, affecting its functional activity by counteracting acetylation modifications. These findings highlight the unexpected tumor-suppressive role of innate immune signaling mediated by TRAF6 in myeloid malignancies 135. Altogether, these studies explain the intricate interplay between dysregulated innate immune pathways and myeloid malignancies.

In conclusion, TRAF6 plays a significant role in myeloid neoplasms, impacting signaling pathways and ubiquitination processes. While it has been associated with promoting tumorigenesis in various cancers, such as glioblastoma 136, nasopharyngeal carcinoma 137, osteosarcoma 138, head and neck squamous cell carcinoma 139, oral cancer 140, its role in myeloid malignancies presents a complex interplay between innate immune pathways and cancer development. Further research is needed to fully explore the specific underlying mechanisms.

## TRAF6 as a therapeutic target in cancers

TRAF6 has the potential to be an effective target in treating cancers; however, the underlying mechanisms of resistance to TRAF6 and radiotherapy have been partially elucidated. Studies have shown increased TRAF6 levels in tumors, with the TRAF6-NF-κB pathway playing a major role. NF-κB activation results in cytokine production, which subsequently induces NF-κB activation in precancerous cells, promoting the abnormal growth and expression of malignancy-associated cells and genes, respectively. When the small-molecule inhibitor 6877002 is used concomitantly with docetaxel, the discernible attenuation of metastasis and a reduction in osteolytic bone damage have been observed in murine models containing 4T1-Luc2 cells 141. Resveratrol reportedly inhibits EMT via the TRAF6/NF-κB/SLUG axis, suggesting that TRAF6 mediates the inhibition of PCa cell proliferation and migration by resveratrol 142. Further, chrysin may inhibit signal transduction and induce TRAF6 degradation by modulating A20-dependent polyubiquitin 143. Additionally, parthenolide suppresses proliferation and induces apoptosis in MM cells by directly binding to TRAF6, thereby inhibiting NF-κB signaling pathway activation 144. The natural small molecule FMHM directly promotes the polyubiquitination of the Lys48 residue by targeting ubiquitin proteins at Lys48. This polyubiquitination subsequently promotes the formation of Lys48-linked polyubiquitin chains on TRAF6 and increases TRAF6 degradation via the UPS, thus ultimately inactivating the downstream NF-κB inflammatory pathway 145. Furthermore, TRAF6 is a characteristic ligase responsible for AKT ubiquitination, followed by AKT recruitment to the cell membrane. Additionally, TRAF6 undergoes phosphorylation in response to stimulation by growth factors. TRAF6 inhibition by RNA silencing or using decoy peptides has the potential to reduce tumor cell proliferation, promote apoptosis, and augment bone resorption in MM. Notably, certain proteasome inhibitors and benzoxadiazole derivatives exert inhibitory effects on TRAF6 activity and function and suggest potential therapeutic strategies 132. Cinchonine can induce apoptosis by inhibiting AKT and TAK1 signaling pathways via competitive binding to the RING domain of TRAF6 146. Moreover, TRAF6 is a relevant target for bortezomib-induced cytotoxicity in myelodysplastic syndrome/acute myeloid leukemia cases, regardless of the chromosome 5q status 134. Epigallocatechin-3-gallate is a novel inhibitor of E3 ubiquitin ligase activity that specifically targets TRAF6, thereby suggesting its potential applications in chemotherapy or melanoma prevention 147. In addition to the aforementioned drug therapies, radiotherapy is a crucial cancer treatment modality; however, relatively few reports are available on radiotherapy targeting TRAF6. The Cox proportional hazards model results suggested that high TRAF6 levels were related to gastric cancer radiosensitivity 148. Chiu et al. explored the potential synergistic effect of combining ionizing radiation (IR) with the proteasome inhibitor MG132 and showed that this combination increased autophagy induction by inhibiting TRAF6, presenting a novel approach for targeting TRAF6 in tumor therapy 85.

## TRAF6 participates in tumor immunity

Tumor immunology is a discipline in which complex interactions between tumor antigens, immune function, and tumor initiation, progression, and outcomes are investigated. It includes the study of immune responses to tumors, strategies used by tumor cells to evade immune surveillance, and the scientific exploration of tumor immunodiagnosis and immunoprevention. TRAF6, a pivotal signaling molecule, exerts regulatory controlling effects on various physiological processes, including adaptive and innate immunity, bone metabolism, and tissue (lymph nodes, mammary glands, skin, and the central nervous system) development. Consequently, the significant involvement of TRAF6 in maintaining immune system homeostasis and affecting tumor immunity necessitates further investigation.

**Figure 3:**
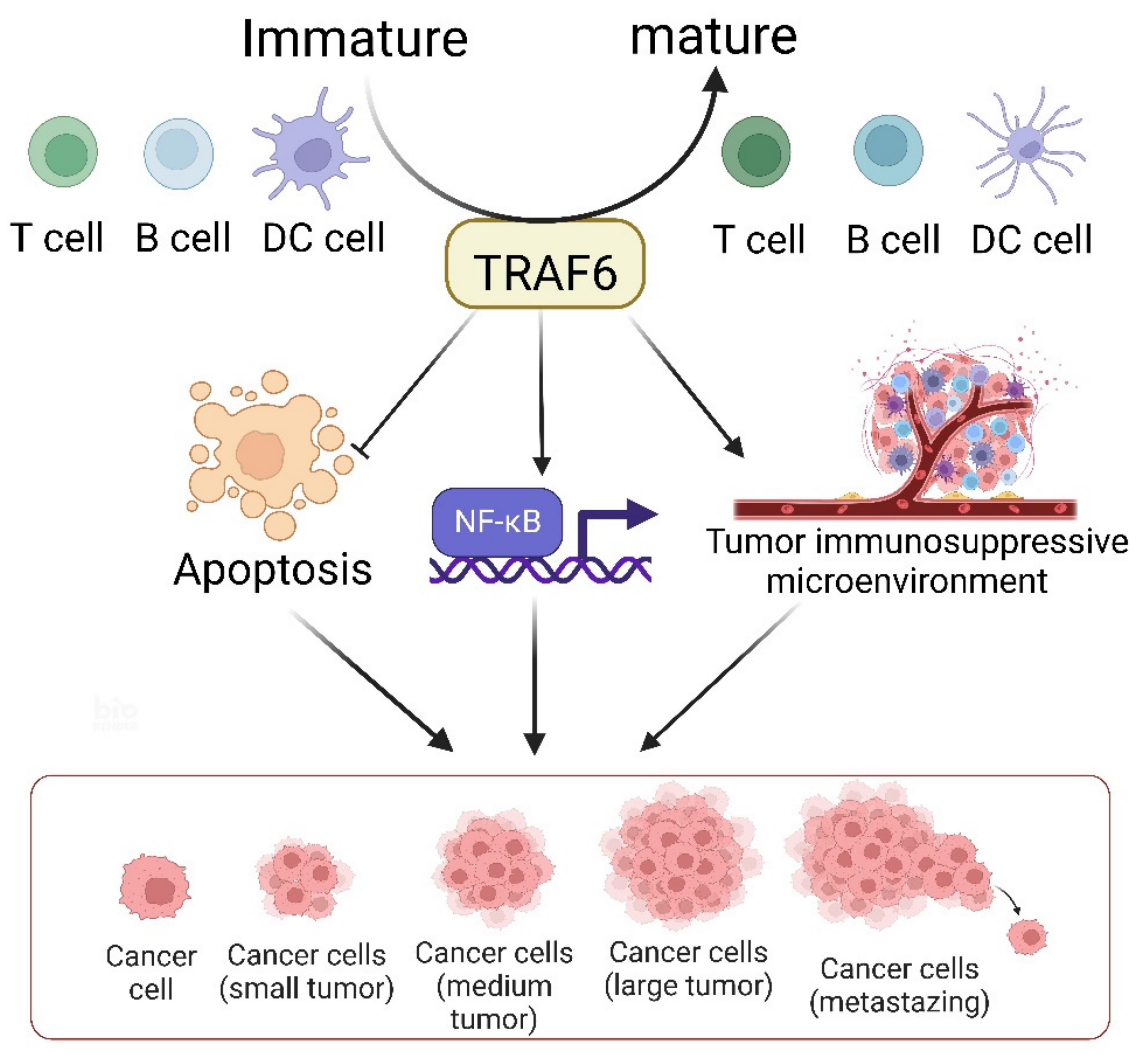
TRAF6 serves as a pivotal determinant in the immune system's control over tumor evolution. It bears multifaceted responsibilities in the biology of dendritic cells (DCs), T cells, and B cells, such as modulating their activation, survival, homeostasis, and tolerance. The tumor microenvironment constitutes an array of cells including macrophages, dendritic cells, neutrophils, mast cells, T cells, and B cells. The generation of diverse inflammatory cytokines, encompassing TNF-α, IL-1, and IL-6, is predicated on the classical NF-κB activation pathway, modulated by inflammation-induced Ikkβ. Secreted cytokines, TNFα and IL-1, exert an impact on precancerous cells by instigating NF-κB activation, which reciprocally facilitates and partakes in the inhibition of cellular apoptosis, the formation of an immunosuppressive tumor microenvironment, and the promotion of cellular proliferation, thereby aiding the evolution of malignant tumors.

The TRAF6-mediated signaling pathway plays a pivotal role in the development, homeostasis, and activation of various immune cells, including B 149, T, and myeloid cells such as macrophages, dendritic cells 150, and osteoclasts. This pathway is indispensable for ensuring proper immune system activation and maintaining immune tolerance across diverse cellular processes and environments (Figure 3). The K63 polyubiquitin pathway, which is associated with TRAF6, is critical in inflammation-driven tumor promotion during carcinogenesis and is closely associated with tumor immunotherapy co-stimulation; the underlying biological mechanisms of malignant cells have been extensively investigated in diverse cancer types. The NF-κB transcription factor family, a well-known protein family for its regulatory roles in the expression of genes crucial for immunity and inflammation 151, serves as a pivotal moiety via which malignant cells evade apoptosis, induce angiogenesis, sustain inflammation, and facilitate metastasis and dissemination. Consequently, the increased level of TRAF6 in tumor cells significantly contributes to cancer promotion by activating the downstream NF-κB signaling cascade via the classical pathway. Additionally, emerging evidence indicates that TRAF6 contributes to improving the immunosuppressive function of myeloid-derived suppressor cells, thus providing a potential target for antitumor immune interventions. Considering the dynamic interplay between cancer cells and their microenvironments, TRAF6 in immune cells play a critical role in cancer progression.

## Conclusions and future perspectives

The multifaceted roles of TRAF6 are expanding with the progress in tumor research. This leads to the question of how a single protein coordinates seemingly contradictory roles in the same cell and affects diverse outcomes downstream of numerous signaling receptors. This complexity warrants further exploration. In summary, TRAF6 functions probably rely on the activity of a single ring finger domain, which affects multiple signaling pathways and plays a crucial role in tumor development. Consequently, drug therapies targeting TRAF6 have been extensively explored, offering a promising basis for developing cancer treatment strategies. Combining TRAF6 inhibition with other treatment modalities, such as immunotherapy, chemotherapy, and targeted therapy, can be the potential option to enhance therapeutic efficacy and overcome drug resistance. Additionally, the development of biomarkers for predicting treatment responses and patient stratification may help develop a more personalized approach to treating cancers.

TRAF6 is pivotal in mediating the polarization of both M1 and M2 macrophages. However, the contribution of tumor-associated macrophages (TAMs) to the modulation of adaptive immune responses and tumor evasion strategies remains a subject of debate. Macrophages regulate their activity and phenotype by integrating signals in the tumor microenvironment (TME). The proinflammatory, immunogenic, and antitumor properties of M1-like macrophages represent opposing ends of a wide spectrum, whereas M2-like macrophages show anti-inflammatory, tolerogenic, angiogenic and protumor effects 152, 153, colorectal cancer 154 and non-small cell lung cancer (NSCLC) 155, although the mechanisms underlying this association are not known. In future studies, it is imperative to elucidate the specific role of TRAF6 in individual macrophages within the tumor microenvironment using single-cell omics techniques. Meanwhile, the current study found that the Hippo/YAP signaling pathway, as a key emerging pathway regulating cell proliferation and apoptosis, has been reported to be involved in the innate antiviral response of macrophages, a process that relies on TRAF6 ubiquitination 156. In addition, on the other hand, we mentioned above that TRAF6 acts on the mTOR signaling pathway by binding to p62. Importantly, mTOR ubiquitination by the p62-TRAF6 complex is central to regulating cell growth, tumor transformation, and autophagy 157. Therefore, targeting TRAF6 to regulate the mTOR signaling pathway may be involved in tumor progression.

Investigations into the relationship between TRAF6 and tumors will raise many questions, the more the exploration, the more the questions. TRAF6 probably contributes to cancer progression via its broader functions, and further research is required to fully understand the diverse roles of TRAF6 in different cancer types and TMEs. This exploration will facilitate the development of specific and effective TRAF6-targeted therapies and the identification of potential synergistic combinations with existing treatments.

## Figures and Tables

**Figure 4:**
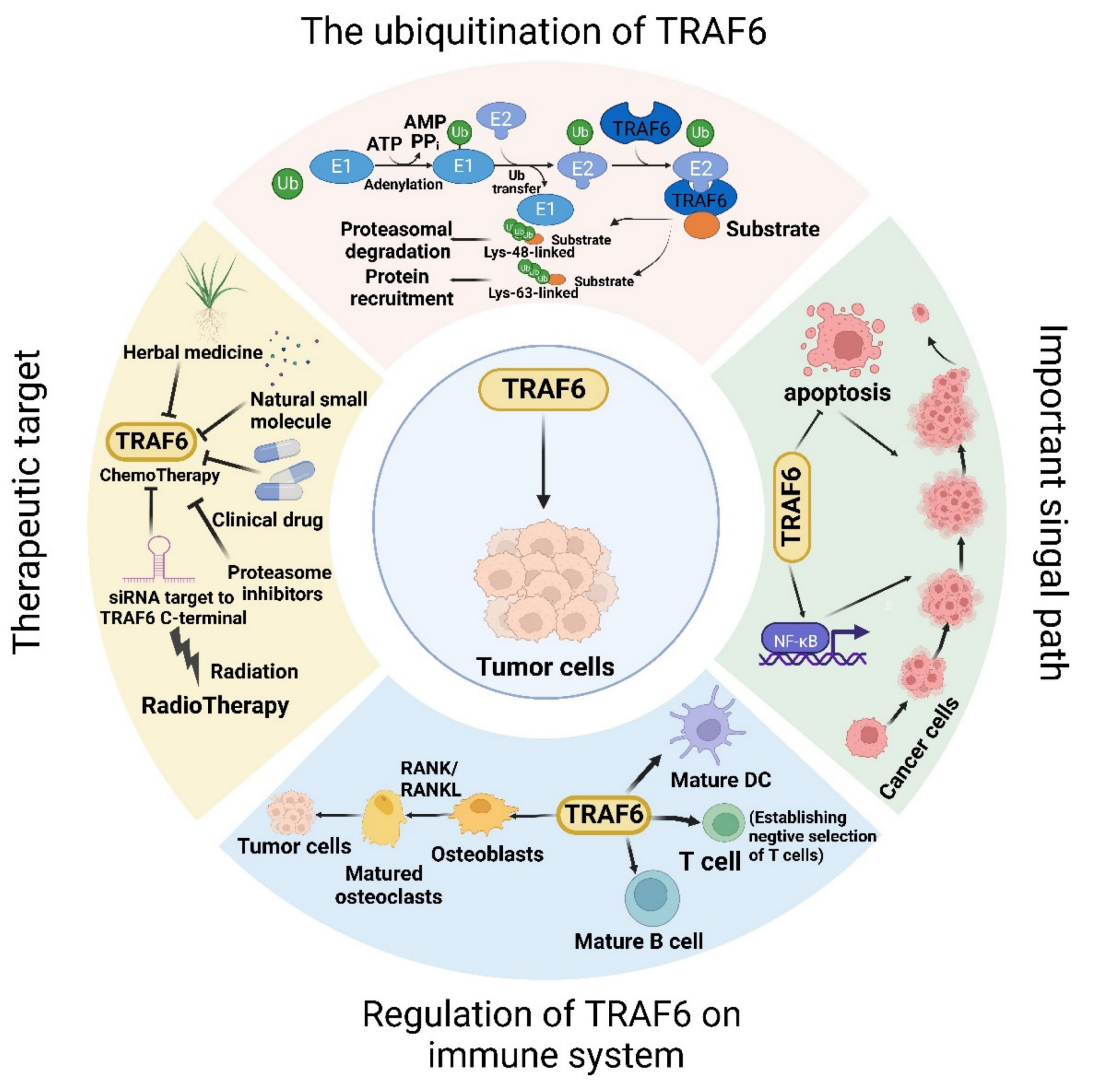
The dynamic involvement of TRAF6 in oncological investigations. Broadly, the function of TRAF6 hinges upon the single ring-finger domain's activity, influencing myriad signaling pathways and playing a consequential role in tumor evolution. The initial step entails TRAF6 ubiquitination, whereby the E1 enzyme establishes a sulfhydryl ester linkage with ubiquitin. The primed ubiquitin is subsequently transferred to E2. TRAF6 (acting as an E3 enzyme) functions as a scaffold for E2 attachment to target entities, facilitating the transference of ubiquitin from E2 to target proteins. Ultimately, TRAF6-mediated polyubiquitination chains linked via lysine are added to the substrate, typically through K48-linked polyubiquitination or K63-linked polyubiquitination. K48-linked polyubiquitin culminates in proteasomal degradation. A single protein harmonizes apparently antithetical signals within cells and impacts various outcomes downstream of numerous signal receptors, particularly by promoting proliferation and inhibiting apoptosis via the stimulation of NF-κB. Moreover, TRAF6 partakes in tumor development through the immune system. Beyond nurturing immune cell maturation, the activation of NF-κB and JNK induced by RANK/RANKL fosters osteoclast maturation and ultimately, tumor cell proliferation. Given its significant role in oncogenesis, TRAF6 is perceived as a promising target for cancer therapeutics, and the mechanism of drug resistance vis-à-vis TRAF6 and radiation therapy has been partially elucidated. In addition to pharmacotherapy, radiation therapy represents a critical modality of cancer treatment.

## Abbreviations

TRAF6: tumor necrosis factor receptor-associated factor 6
TME: the tumor microenvironment
NF-κB: nuclear factor kappa-light-chain-enhancer of activated B
iTME: inflammatory tumor microenvironment
HNSCC: head and neck squamous cell carcinoma
UBD: ubiquitin binding domain
TNFR: tumor necrosis factor receptor
MALT1: mucosaassociated lymphoid tissue lymphoma translocation gene 1
BCL10: B-cell CLL/lymphoma 10
CARMA1: caspase recruitment domain family, member 11
IKKγ: inhibitory κB kinase
TAK1: TGF-β-activated kinase 1
MAPK: mitogen-activated protein kinase
ERK: extracellular signal-regulated kinase
JNK: C-Jun N-terminal kinase
PI3K: phosphatidylinositol 3-kinase
AP-1: activator protein-1
GSK-3β: glycogen synthase kinase-3β
CRC: colorectal cancer
AP: acute pancreatitis
PDAC: Pancreatic ductal adenocarcinoma
ERK1/2: extracellular signal-regulated kinase 1/2
NSCLC: non-small cell lung cancer
TLR4: Toll-like receptor 4
IRF7: interferon regulator 7
KDM4B: jumonji domain containing 2B
IGF-1: insulin-like growth factors
IBD: inflammatory bowel disease
CAC: colitis-associated colon cancer
STX2: Recombinant Syntaxin 2
TRIM25: tripartite motif-containing 25
EZH2: enhancer of zxeste homolog 2
CTNNB1: Catenin Beta 1
EMT: epithelial-mesenchymal transition
MAP1LC3B: microtubule-associated protein 1 light chain 3 beta
NLRC3: NLR family, CARD domain containing 3
PGE2: prostaglandin E2
PTGES2: prostaglandin E synthase 2
GC: gastrointestinal cancer
FOXO: forkhead box O
IL-17: interleukin-17
TNM: tumor-node-metastasis
CagA: cytotoxin-associated gene A protein
MEKK3: mitogen-activated protein kinase kinase 3
HR: homologous recombination
DSB: DNA double-strand break
TMEM189: transmembrane protein 189
ULK1: Unc-51 like autophagy activating kinase 1
MIP-3A: macrophage inflammatory protein-3A
HCG18: human chorionic gonadotropin 18
PC: Pancreatic cancer
USP4: ubiquitin-specific proteinase 4
YAP: yes-associated protein
TAZ: transcription coactivator
CYB5A: cytochrome b5 A
EC: esophageal carcinoma
AEP: asparaginyl endopeptidase
MMP2: matrix metalloproteinase 2
EAC: esophageal adenocarcinoma
ESCC: esophageal squamous cell carcinoma
PBMC: peripheral blood mononuclear cells
HCC: hepatocellular carcinoma
HBV: hepatitis B virus
HCV: hepatitis C virus
NAFLD: non-alcoholic fatty liver disease
SNHG16: small nucleolar RNA host gene 16
HBx: Hepatitis B virus X
GADD34: growth arrest and DNA damage-inducing gene 34
TRAIL: tumor necrosis factor-associated apoptosis-inducing ligand
MCL-1: myeloid leukemia 1
TRIM59: tripartite motif-containing 59
RCC: renal cell carcinoma
ccRCC: clear cell renal cell carcinoma
TP53INP2: tumor protein p53 inducible nuclear protein 2
SKP2: S-phase kinase-interacting protein
DRAK1: death-related protein kinase-associated apoptosis-inducing kinase 1
TNBC: triple-negative breast cancer
MM: multiple myeloma
CML: chronic myeloid leukemia
EGCG: epigallocatechin-3-gallate
MDSC: myeloid-derived suppressor cells
